# Risk factors for small bowel obstruction after open rectal cancer resection

**DOI:** 10.1186/s12893-021-01072-y

**Published:** 2021-01-28

**Authors:** Kevin Afshari, Abbas Chabok, Kenneth Smedh, Maziar Nikberg

**Affiliations:** Colorectal Unit, Department of Surgery and Centre for Clinical Research of Uppsala University, Västmanland’s Hospital Västerås, 72189 Västerås, Sweden

**Keywords:** Small bowel obstruction, Rectal cancer, Surgery, Admission, Risk factors

## Abstract

**Background:**

Small bowel obstruction (SBO) is observed in around 10% of patients with prior open abdominal surgery. Rectal resection causes the highest readmission rates. The aim of this study was to investigate risk factors for readmission for SBO and causes for SBO in patients who needed surgery following rectal cancer surgery.

**Methods:**

A population-based registry with prospectively gathered data on 752 consecutive patients with rectal cancer who underwent open pelvic surgery between January 1996 and January 2017 was used. Univariable and multivariable regression analysis was performed, and the risk of SBO was assessed.

**Results:**

In total, 84 patients (11%) developed SBO after a median follow-up time of 48 months. Of these patients, 57% developed SBO during the 1st year after rectal cancer surgery. Surgery for SBO was performed in 32 patients (4.3%), and the cause of SBO was stoma-related in one-fourth of these patients. In the univariable analysis previous RT and re-laparotomy were found as risk factors for readmission for SBO. Re-laparotomy was an independent risk factor for readmission for SBO (OR 2.824, CI 1.129–7.065, *P* = *0.026*) in the multivariable analysis, but not for surgery for SBO. Rectal resection without anastomoses, splenic flexors mobilization, intraoperative bleeding, operative time were not found as risk factors for SBO.

**Conclusions:**

One-tenth of rectal cancer patients who had open surgery developed SBO, most commonly within the 1st postoperative year. The risk of SBO is greatest in patients with complications after rectal cancer resection that result in a re-laparotomy.

## Background

Abdominal surgical trauma causes adhesion in almost all patients [1]. Although the majority of patients are asymptomatic, a significant number suffer from small bowel obstruction (SBO) [2], female infertility [3], and chronic pain [4]. After abdominal surgery, SBO has been observed in 9% of patients [5]. A readmission rate of 30% during a 10-year period [6] has been reported, and most cases occur within in the 1st year after abdominal surgery [7]. Rectal cancer surgery causes the highest readmission rates [5, 8, 9].

Several studies have focused on outcomes in adhesion-related SBO. Mortality rates in SBO surgery are reported to be up to 10% [10], rising to 15% [11], when small bowel resection is performed, with a 33% risk of inadvertent enterotomy during surgery for bowel obstruction [12].

Surgery for rectal cancer has the highest adhesion-related readmissions; therefore, it is important to elucidate the risk factors for SBO related to rectal cancer surgery. Some surgeons believe that omentoplasty to fill the pelvis lowers the risk of SBO. An empty pelvis after abdominoperineal excision (APE) or Hartmann’s procedure (HP) may also lead to a higher risk of SBO compared with anterior resection (AR). However, results from studies are not consistent [13].

There are few studies on rectal cancer and many studies are retrospective, using ICD-10 codes to identify patients and focus only on adhesion-related SBO. The aim of the present study was to investigate risk factors for SBO readmission and the different causes of SBO in patients with prior open rectal cancer surgery, in a cohort where data has been collected prospectively.

## Methods

This study was based on prospectively collected data from a local population-based registry on all openly operated patients with rectal cancer diagnosed between January 1996 and January 2017 in Västmanland county. This comprehensive local data set includes detailed pre-, peri-, and postoperative data, as well as follow-up data with information on admission for SBO with or without surgery. Patients were scheduled for follow-up at 1, 6, 12, 24, 36, 48, and 60 months after surgery.

Rectal cancer was identified with a rigid rectoscope and defined as an adenocarcinoma with a distal margin within 15 cm from the anal verge.

### Study population

In total, 1136 patients were diagnosed with rectal cancer during this period. Of these, 159 did not undergo any intervention, 191 underwent interventions such as endoscopic resection, polypectomy, stoma formation, or stent placement and 34 underwent laparoscopic surgery (starting from 2015). In total, 752 underwent open rectal tumor resection who were included in the study.

### Data variables

All data were collected prospectively at each follow-up. In patients with SBO during the follow-up period, additional data were retrieved from medical records, and supplementary information was gathered from the surgical platform to identify those patients who had been admitted and treated without or with surgery for SBO. For all patients who underwent surgery for their SBO the cause of obstruction was registered after surgical notes were scrutinized.

In 60 patients, follow-up data were collected retrospectively from their medical records because of missing data in the registry. Patients were noted as lost to follow-up if they had died or moved outside the county. In the case of death, data were collected through the Swedish Death Registry.

Preoperative screening for metastases was routinely performed. Up until 2002, chest radiography and liver ultrasonography were used, and thereafter, computed tomography of the thorax and abdomen. Magnetic resonance imaging of the pelvis was used for local staging in almost all cases. Stage was defined according to the 6th American Joint Committee of Cancer (AJCC) TNM classification.

### Definitions

SBO was defined as any admission to the hospital or surgery, due to small bowel obstruction occurring after 30 days postoperatively, diagnosed with radiography. Early post-operative bowel paralysis was not registered as SBO but rather as a post-operative complication.

Patients with clinical signs of SBO (abdominal pain, vomiting, distention) were diagnosed on plain abdominal X-ray or computed tomography (CT). Conservative treatment involved nasogastric tube, intravenous fluids for resuscitation and serial X-rays for diagnostic and therapeutic purposes. In patients with contrast passage to the colon at the abdominal radiography, the nasogastric tube was removed. If the contrast failed to reach the colon or if the patient’s condition deteriorated, patients underwent surgery. Surgical treatment involved exploratory laparotomy with adhesiolysis, with or without small bowel resection.

The scoring system of the American Society of Anesthesiologists (ASA) was used to determine a patient’s degree of comorbidity, and performance status was graded according to World Health Organization performance status (WHO).

Based on the type of surgery, two groups where created: resection with and without anastomosis. Resection with anastomosis included AR and patients who had procto-colectomy with pelvic reservoir. The group without anastomosis included HP and APE.

Surgical details of the rectal cancer surgery have been described previously [14, 15]. The omentum majus was placed along with the descending colon, but no omentoplasty was performed to fill the pelvis in any patient. Adhesion preventive solutions were not used. Resection of other organs was defined as an en bloc resection of adjacent organs in locally advanced rectal tumors. This cohort included all resected rectal tumors both curatively intended and palliatively resected. Postoperative surgical complications within 30 days were anastomotic leakage, infected hematoma, pelvic abscess, colovaginal fistula, bleeding, abdominal wall dehiscence, and stoma complications. Other risk factors of interest were the amount of surgical trauma, including minimal invasive surgery, mobilization of splenic flexure, and re-laparotomy due to complications. Incisional hernia was observed either through a clinical examination during follow-up or using computer tomography. Radiotherapy (RT) included both patients who had undergone RT because of rectal cancer or because of other previously treated pelvic tumors.

### Statistical analysis

Continuous data were reported as mean ± standard deviation (SD) or median with range. Categorical data were analyzed for differences in proportions using the χ^2^-test or Fisher’s exact test for low numbers. Univariable and multivariable analyses for factors affecting admission or surgery for SBO were performed using binary logistic regression. The multivariable analyses used all possible factors affecting SBO admission. For incisional hernia, Spearman correlation was used. *P* values < 0.05 were considered statistically significant. Data were analyzed using SPSS software (v. 24; IBM Corp., Armonk, NY, USA).

## Results

A total of 752 patients underwent open rectal cancer resection between 1996 and 2017 and were included in the study. Of those, 733 patient (97.5%) were followed up for more than 6 months, and 19 patients (2.5%) died before the 6-month follow-up. The median follow-up time was 48 months (range: 0–96 months).

The patients’ characteristics are summarized in Table 1. The mean age was 68 ± 10 years. Sixty-two percent were male, and the majority were ASA 1–2. Most patients had a WHO performance status of 0–1. Sixty-two percent of the patients underwent AR, and the splenic flexure was mobilized in 60%. In total, 579 patients (77%) had RT, whereof 18 patients (2.4%) had received RT previously for prostatic or gynecological cancer.

**Table 1:** Demographics, patient, and surgical characteristics of patients undergoing surgery for rectal cancer in Västmanland county between 1996 and 2017

Parameters	Total cohort *N* = 752	With SBO *N* = 84	Without SBO *N* = 668
Age (years)^b^	69 (32–88)	66 (42–87)	70 (32–88)
Gender M: F	469: 283(62:38)	53:31(63:37)	416:252(62:38)
Body mass index (kg/m^2^)^c^	26 ± 4	25 ± 4	25 ± 4
Missing	5	0	5
ASA score			
I–II	524 (70)	61 (73)	463 (69)
III–IV	228 (30)	23 (27)	205 (31)
WHO PS			
I	435 (58)	51 (61)	384 (57)
II	282 (37)	30 (36)	252 (38)
III	28 (4)	3 (3)	25 (4)
IV	6 (1)	0 (0)	6 (1)
TNM stage			
I–II	404 (54)	42 (50)	362 (54)
III	256 (34)	29 (35)	227 (34)
IV	91 (12)	13 (15)	78 (12)
Preoperative radiotherapy^a^	579 (77)	73 (87)	506 (76)
Missing	1	0	1
Type of surgery			
Anterior resection	468 (62)	48 (57)	420 (63)
Hartmann’s procedure	84 (11)	7 (8)	77 (11)
Abdominoperineal excision	184 (25)	27 (32)	157 (24)
Proctocolectomy	16 (2)	2 (3)	14 (2)
Mobilization of splenic flexure	454 (60)	51 (61)	403 (60)
Resection of other organs	202 (27)	25 (30)	177 (27)
Intraoperative bleeding (mL)^c^	689 ± 618	723 ± 604	685 ± 620
Missing	3	0	3
Operative time (min)^c^	293 ± 76	302 ± 70	292 ± 76
Missing	5	0	5
Re-laparotomy	28 (4)	7 (8)	21 (3)
Incisional hernia	69 (9)	10 (12)	59 (9)

Other values in parentheses are percentages

*SBO* Small bowel obstruction, *ASA* American Society of Anesthesiologists, *WHO PS* World Health Organization performance status

^a^Preoperative radiotherapy is any radiotherapy given at any time prior to surgery for rectal cancer, including radiotherapy for cancers other than rectal cancer

^b^Values are median (range)

^c^Continuous values are presented as mean ± standard deviation

Surgical complications were seen in 158 patients (21%) (Table 2) and in 28 patients the surgical complications resulted in a re-laparotomy. Re-laparotomy due to complications were associated with an increased risk of admission for SBO both in the univariable (OR 2.853, CI 1.174–6.936, P = 0.021) and multivariable analysis (OR 2.824, CI 1.129–7.065, P = 0.026) (Table 3). Previous RT was associated with increased risk of hospital admissions for SBO in the univariable analysis (OR 2.112, CI 1.093–4.078, P = 0.026) but not in the multivariable analysis (Table 3).

**Table 2:** Total number of complications after rectal cancer surgery in Västmanland county between 1996 and 2017 and complications leading to re-laparotomy

Complications	N = 158	Re-laparotomy (N = 28)
Anastomotic leakage	31 (20)	10
Abscess in the lower abdomen	10 (6)	1
Bleeding	1 (1)	1
Abdominal wall dehiscence	3 (2)	3
Stomal complications	10 (6)	3
Intestinal injury	4 (2)	4
Ileus	9 (6)	4
Wound infection	46 (29)	
Perineal wound infection	42 (27)	
Colovaginal fistula	2 (1)	
Other		2^a^

Values in parentheses are percentages

^a^Two patient had normal finding at laparotomy

**Table 3:** Univariable and multivariable logistic regression analysis of patients undergoing surgery for rectal cancer in Västmanland county between 1996 and 2017 who were admitted for small bowel obstruction (SBO)

Admission for SBO	Univariable			Multivariable		
	OR	95% CI	*P*	OR	95% CI	*P*
Age	0.981	0.961–1.002	0.082	0.981	0.958–1.005	0.113
Gender						
Female	1		0.884	1		0.941
Male	1.036	0.647–1.657		1.019	0.616–1.688	
Body mass index	0.975	0.920–1.034	0.404	0.969	0.910–1.031	0.321
ASA score						
I–II	1		0.535	1		0.762
III–IV	0.852	0.513–1.414		0.913	0.505–1.648	
TNM stage						
I–II	1			1		
III	1.101	0.667–1.818	0.707	1.148	0.682–1.932	0.603
IV	1.437	0.736–2.803	0.288	1.346	0.660–2.743	0.414
Preoperative radiotherapy^a^						
No	1		0.026	1		0.071
Yes	2.112	1.093–4.078		1.932	0.945–3.951	
Type of surgery						
Resection with anastomosis	1		0.321	1		0.225
Resection without anastomosis	1.262	0.797–1.999		1.614	0.745–3.495	
Mobilization of splenic flexure						
No	1		0.946	1		0.271
Yes	1.016	0.639–1.617		1.519	0.722–3.197	
Resection of other organs						
No	1		0.525	1		0.832
Yes	1.175	0.714–1.935		0.938	0.519–1.696	
Intraoperative bleeding	1	1.000–1.000	0.596	1	1.000–1.000	0.853
Operative time	1.002	0.999–1.005	0.232	1	0.996–1.004	0.865
Re-laparotomy						
No	1		0.021	1		0.026
Yes	2.853	1.174–6.936		2.824	1.129–7.065	

*ASA* American Society of Anesthesiologists, *CI* confidence interval, *OR* odds ratio

^a^Preoperative radiotherapy is any radiotherapy given at any time prior to surgery for rectal cancer, including radiotherapy for cancers other than rectal cancer

SBO was diagnosed in 84 patients (11%), and in 48 patients (57%), SBO occurred during the 1st postoperative year. Surgery for SBO was performed in 32 patients (4.3%) admitted during the study period. The 30-day mortality after surgery for SBO was 3% (*N* = 1), and the 90-day mortality was 9% (*N* = 3).

In 24 patients of the 32 patients (75%) who had surgery for SBO, adhesions were observed in the abdominal wall (*N* = 10), in the pelvis (*N* = 7), and in both the abdominal wall and pelvis (*N* = 7). The cause of SBO was in addition to adhesions, related to the stoma in 9 patients (28%); stenosis before closure of the loop ileostomy (*N* = 2), strangulation of the small bowel around the loop ileostomy (*N* = 3), and stenoses after closure (*N* = 4). The remaining causes of SBO were abdominal malignancy (*N* = 4), such as carcinomatosis or recurrent tumor, and unspecified in two.

Morbidity, age, type of rectal cancer surgery (i.e., with or without anastomosis), more extensive surgical dissection planes, such as mobilization of the left flexure and resection of other organs, were not associated with the rate of hospital admissions (Table 3). Of those patients who underwent an AR, 74% received a loop ileostomy with no increased risk of admission due to SBO (*P* = 0.569, data not shown). None of the analyzed factors resulted in an increased risk of surgical outcome for SBO in the logistic regression analysis (data not shown). Sixty-nine patients developed incisional hernia, and no correlation was found between admission and surgery for SBO and incisional hernia (data not shown).

## Discussion

The long-term risk of SBO after rectal cancer surgery was 11%, and most patients were admitted in the 1st postoperative year. Surgery for SBO was performed in 4.3% of patients, and the causes were related to adhesions in 75% and some in addition had SBO related to their stoma. Patients who had previous RT had a twofold increased risk of SBO necessitating hospital admission and patients with postoperative complications that resulted in re-laparotomy had a threefold increased risk for future admission due to SBO. However, the type of rectal cancer surgery (i.e., with or without anastomosis) or more extensive surgical dissection were not associated with hospital admission rates or surgery for SBO.

We present one of the largest prospectively collected cohorts on rectal cancer patients who had open surgery and the risk of admission due to SBO. Our data are in accordance with a previously reported study based on a Swedish inpatient registry [9]. The risk of surgery for SBO has been studied extensively. One study based on data from a Danish registry found this risk to be 4.5% [16], which is the same as that in the present study. Another study found the risk to be between 19 and 66% [17]. In addition, the increased risk of SBO during the 1st year after surgery (57%) is in concordance with previous studies that report risks of 22–60% [6, 7, 18].

Within the cohort, we report a high rate of loop ileostomy-related SBO with various causes both before and after closure, which together with other negative ileostomy-related outcomes, such as poorer functional outcomes and costs, raises concern about the routine use of loop ileostomy [19, 20].

Stoma formation is a risk factor for SBO in colorectal surgery, but no consensus has been reached regarding whether the type of stoma affects the SBO outcome. One study found an increased risk of SBO in colorectal surgery in those with stoma, but no difference in the incidence of SBO was found between ileostomy or colostomy [21].

Gastrointestinal dysfunction, especially bowel obstruction, is well known after RT [22, 23]. In the Swedish Rectal Cancer Trial [22], an approximate twofold increased risk of bowel obstruction was shown as both an early and late adverse effect of RT. This was also found in this study in the univariable analyses for admission due to SBO however not in the multivariable analysis and not for surgery for SBO. This is probably because of different study periods, differences in radiation fields or due to type II error.

Interestingly, the type of rectal cancer surgery with regard to resection with and without anastomosis or the extent of dissection was not a risk factor for admission and surgery for SBO. However, surgical complications that resulted in re-laparotomy after rectal cancer surgery were an independent risk factor for admission for SBO. One can speculate that this is probably partly due to peritoneal inflammation related to the complications, and partly to secondary surgical trauma, which, in turn, causes additional adhesions.

To reduce the risk of ileus, omentoplasty is performed by some surgeons to prevent the decent of the small bowel in the empty pelvic cavity after APE and HP, even though, to our knowledge, without any evidence of reducing this risk. On the contrary, a recent study reported that the readmission and reintervention rates for SBO did not differ, with or without omentoplasty [13]. In the present cohort, none of the patients had an omentoplasty, and we could not find any difference regarding SBO admissions between those with anastomosis, i.e., filled pelvis after AR, versus those without an anastomosis, i.e., after APE and HP.

The main strength of this study is its population-based design with prospectively registered data, which limits the bias associated with retrospective studies. The cohort was homogenous, with only rectal cancer patients operated with the intent of local radical resection. As this was a single center study, the surgery was standardized, and only few colorectal surgeons operated on the patients. All variables were registered at each follow-up visit by the same surgeons, and consensus was reached on the definitions of all variables. For patients with SBO identified in the registry, medical records were scrutinized and etiologies behind SBO were identified.

The results should though be interpreted cautiously because of the low number of patients with SBO. In the present study, we included patients regardless of previous surgery before the index surgery, which may have introduced bias.

## Conclusion

One-tenth of rectal cancer patients who had open surgery developed SBO during follow-up, most commonly within the 1st postoperative year. The risk of SBO is greatest in patients with complications after rectal cancer resection that result in a re-laparotomy. The type of resection surgery, with or without anastomosis, was not a risk factor for SBO.

## Data Availability

The datasets used and/or analysed during the current study are available from the corresponding author on reasonable request.

## Abbreviations

AJCC: American Joint Committee of Cancer
ASA: American Society of Anesthesiologist
APE: Abdominoperineal excision
AR: Anterior resection
HP: Hartmann’s procedure
SBO: Small bowel obstruction
WHO: World Health Organization
RT: Radiotherapy
